# AntiTbPdb: a knowledgebase of anti-tubercular peptides

**DOI:** 10.1093/database/bay025

**Published:** 2018-02-28

**Authors:** Salman Sadullah Usmani, Rajesh Kumar, Vinod Kumar, Sandeep Singh, Gajendra P S Raghava

**Affiliations:** 1 Bioinformatics Centre, CSIR-Institute of Microbial Technology, Sector 39A, Chandigarh - 160036, India; 2 Centre for Computational Biology, Indraprastha Institute of Information Technology, Okhla, New Delhi - 110020, India

## Abstract

Tuberculosis is a global menace, caused by *Mycobacterium tuberculosis*, responsible for millions of premature deaths every year. In the era of drug-resistant tuberculosis, peptide-based therapeutics may provide alternate to small molecule based drugs. In order to create knowledgebase, AntiTbPdb (http://webs.iiitd.edu.in/raghava/antitbpdb/), experimentally validated anti-tubercular and anti-mycobacterial peptides were compiled from literature. We curate 10 652 research articles and 35 patents to extract anti-tubercular peptides and annotate these peptides manually. This knowledgebase has 1010 entries, each entry provides extensive information about an anti-tubercular peptide such as sequence, chemical modification, chirality, nature and source of origin. The tertiary structure of these anti-tubercular peptides containing natural as well as chemically modified residues was predicted using PEPstrMOD and I-TASSER. In addition to structural information, database maintains other properties of peptides like physiochemical properties. Numerous web-based tools have been integrated for data retrieval, browsing, sequence similarity search and peptide mapping. In order to assist wide range of user, we developed a responsive website suitable for smartphone, tablet and desktop.

**Database URL**: http://webs.iiitd.edu.in/raghava/antitbpdb/

## Introduction

Tuberculosis (TB) is one of the deadliest diseases of mankind caused by *Mycobacterium tuberculosis* (*M. tuberculosis*). According to WHO report 2016, TB infects 10.4 million people worldwide and cause the death of 1.8 million people in year 2015. This put the TB as a second world deadliest disease after HIV/AIDS. The initial anti-TB drugs isoniazid, streptomycin introduced some 50 years ago, led to the optimization that TB could be eradicated. Due to this inception in mind, pharmaceutical industry develops only few drugs in the past few years for the treatment of TB. Since 1980 s the TB has undergone a revivification impelled by a number of factors like increase in immunosuppressed Patient (1). Current TB treatment strategy is far from satisfactory as overall treatment duration is long, approximately equal to 12 months (2) as well as requires daily administration of drugs, which are toxic and less effective in case of multi-drug or extensive drug resistance TB as delineated by its high mortality rate (∼15%). There is a need of hour to discover or design effective drugs against tuberculosis, particularly against multi-drug resistance (MDR-TB), extensive drug resistance (XDR-TB) and emerging extreme drug resistance strain (XXDR-TB) (3).

Due to continuous failure of antibiotics against drug resistant bacteria, pharmaceuticals industries are looking for alternative strategy. One of the possible alternatives to antibiotics (traditional drug mainly based on small molecules) is peptide based therapeutics. A significant and vast majority of peptides from plant, bacteria and fungus sources proven to have anti-microbial action and these can be used as a supplement or alternate for conventional antibiotics (4). In last few decades, researchers have screened several peptides effective against *Mycobacterium*, which has been used for TB treatment in different therapeutic strategy like as single anti-TB agents (5), in combination with conventional drugs (6) and synergistic effect with traditional antibiotic therapy (7).

The emergence of anti-tubercular peptide as a promising therapeutic candidate is due to its specific molecular action as selective affinity towards cell envelope, low toxicity, low immunogenicity and targeted immune response against invading pathogens (8, 9). Several anti-tubercular peptides have shown promising results in various pre-clinical studies and their scattered information in literature is difficult to access. The huge therapeutic importance of anti-TB peptides, motivate us to make a database fully dedicated to anti-tubercular or anti-mycobacterial peptides. Several mutant protein or protein complex databases (10, 11), peptide database of therapeutic importance (12–18) as well as anti-microbial database (19–23) covering some anti-mycobacterial peptides exists, but to the best of our knowledge, no single repository fully dedicated to anti-tubercular or anti-mycobacterial peptide database exist, till date.

## Materials and methods

### Data acquisition

Anti-tubercular peptides were manually collected from research articles and patents. Combination of keywords like ‘anti-tubercular peptide’, ‘anti-tuberculosis peptide’, ‘anti-mycobacterial peptides’ and ‘anti-microbial peptides against *M.tuberculosis’* in PubMed search criteria resulted into 10 652 articles. All the articles were manually screened for relevant experimental information, and ∼900 articles were filtered. Further reviews and articles lacking relevant information were excluded and final data were curated from 96-research article. Similarly, patents were searched from USPTO with same keywords and resulted into 35 patents, and after careful reading data from 5 patents, which were openly available in native English language, were curated.

### Database architecture and web interface

**Figure 1.**
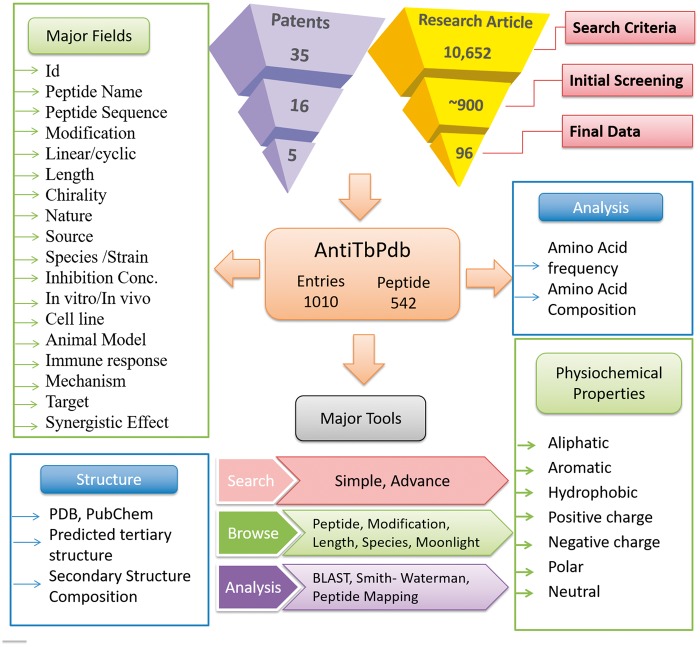
A schematic representation of information organization in AntiTbPdb.

AntiTbPdb is built on Apache HTTP server (version 2.2.17), which is installed on machine with Ubuntu as operating system. The responsive front-end, which is suitable for mobile, tablet and desktop, was developed using HTML5, CSS3, PHP5 and JavaScript. MySQL (a relational database management system, version 5.0.51 b) was used at the back-end to manage the data. The architecture of AntiTbPdb is shown in Figure 1.

### Database organization

Broadly, the data in this repository or database can be divided in two categories namely primary and secondary. Primary data includes manually curated information from research articles and patents like peptide name, its sequence, end terminal as well as chemical modification, its nature and source of origin. Beside this *Mycobacterium* species and its corresponding strain, cell lines and animal model that have been used to check peptides efficacy, inhibition concentration and cytotoxicity are also maintain under this category. We have also carefully looked-for peptide’s immune response directed against anti-TB peptides and wherever the information is available has been included in the database.

Secondary data includes the information derived from primary data such as physiochemical properties, amino acid compositions and frequency of anti-tubercular peptides. In-house PERL scripts have been used to calculate frequency, composition and physiochemical properties. Since structure plays an important role while elucidating peptides function, therefore we have also annotated structure of most of the peptides stored in AntiTbPdb. Following strategies have been adopted for extracting structure of peptides from existing resources and for predicting structure of peptides. Firstly, existing repositories of experimentally determined structures such as PDB or PubChem were examined for assigning structure of anti-tubercular peptides. Secondly, peptide structure prediction method PEPstrMOD (24) was used for remaining peptides whose sequence length is up 25 amino acids. PEPstrMOD predicts both natural as well as modified amino acids like ornithine, N-methyl alanine etc. Finally, we used I-TASSER (25) for predicting structure of remaining peptides. 

### Data retrieval tools

#### Searching

This module of AntiTbPdb has been designed to facilitate easy searching of data using simple and advanced search options. In simple search module, the user can give the query against any filed of the database such as name, sequence, inhibition concentration, *Mycobacterium* species, N or C-terminal modification, chemical modification, mechanism of action, target and PMID or patent number. This option allows the output customized according to the search query. In the advanced search module, the user can give multiple queries simultaneously with Boolean expressions (e.g. AND, OR and NOT).

#### Browsing

A user-friendly browsing interface has been developed to facilitate easy retrieval of the information. We have computed physicochemical properties of each anti-tubercular peptide such as the hydrophobicity, aliphaticity, aromaticity, polar or neutral charge, negative or positive charge. Users can browse peptides entries for the desired physicochemical properties. Various cross-linked browsable tables have been provided to easily access the data. The users can browse on major fields: (i) peptides (e.g. Peptide type, Source, Chirality and nature) (ii) modification of peptides (e.g. N- or C-terminal modification, chemical modification), (iii) length of peptide, (iv) *Mycobacterium* species (classified as pathogenic, non-pathogenic and opportunistic) and (v) moonlight; many peptides have shown bactericidal activity against more than one *Mycobacterium* species, for the easiness of users, they can be browsed under moonlight category.

#### Composition

In addition to the information on anti-tubercular peptide sequences, the most relevant physicochemical properties of peptides are calculated like charge, hydrophobicity, amphipathicity, aromaticity, etc. This is a very important analysis tool, which helps user to analyse and retrieve peptide with desired amino acid composition/frequency and physicochemical properties. This tool has five modules: (i) amino acid (AA) composition, (ii) AA frequency, (iii) physicochemical property (PP) composition, (iv) PP frequency and (v) secondary structure (SS) composition. SS composition module assists user to explore anti-tubercular peptides based on their secondary structure composition. For example, using this tool, user can retrieve all anti-tubercular peptide, which have high composition of turn or helical state. 

#### Sequence alignment

In pursuance of sequence-similarity based search, we have integrated several alignment-based web tools. These similarity-based search tools include BLAST and Smith-Waterman algorithm. Users have to submit their protein or peptide in FASTA format with desired or default parameters of BLAST (26). The server implements BLAST search against primary structure or sequence of all the peptides stored in the database, for query sequence. Furthermore, Smith–Waterman algorithm (27) has also been integrated. In addition to sequence-similarity based search, sequence-mapping based on identical residues is also implemented in the webserver i.e. sub-search and super-search. Sub-search module could be used for mapping query peptide against all the peptides deposited in AntiTbPdb, whereas super-search allows peptide mapping and identification of segments that are identical to peptides stored in AntiTbPdb.

## Results

**Figure 2.**
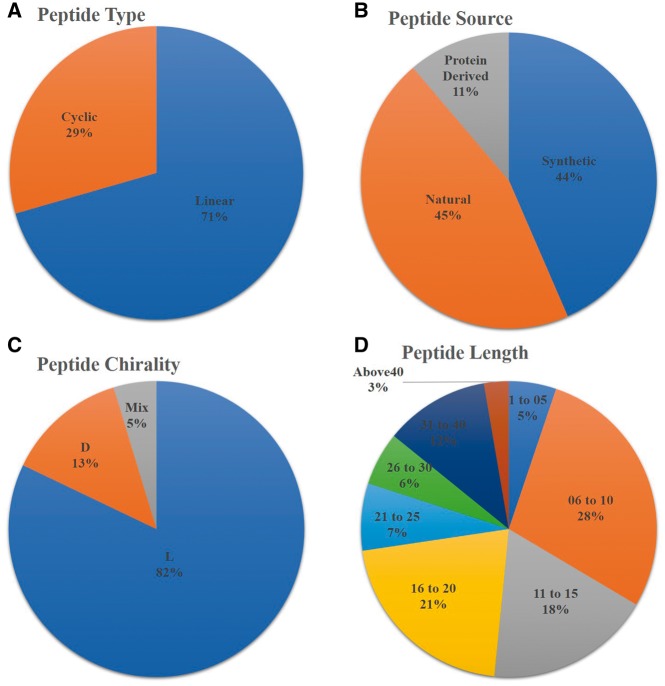
Statistical distribution of peptides in AntiTbPdb based on (**A**) linear/cyclic confirmation, (**B**) source, (**C**) chirality and (**D**) length of peptide.

AntiTbPdb is a unique repository of experimentally verified anti-tubercular or anti-mycobacterial peptides. It contains 1010 entries of 542 unique peptides. It covers diverge range of anti-mycobacterial or anti-tubercular peptide entries that include linear (668), cyclic (279), entry of peptides with L- amino acids (771), D-amino acids (124) and having both L and D amino acids (44). Most of the entries of anti-tubercular peptides are screened from natural sources (434) or derived from specific proteins (112). Its analysis revealed that most of entries of anti-tubercular peptides (672) are shorter in length ranging from 06 to 20 amino acids but some peptide fragments derived from proteins (ex RNase) are even having 70 amino acids (Figure 2). These peptides are also classified based on their nature like cationic, amphipathic, basic etc. In AntiTbPdb, around 619 entries of cationic and 51 show amphipathic nature. Beside this, many are hydrophobic (67) and basic (19) in nature as well.

**Figure 3.**
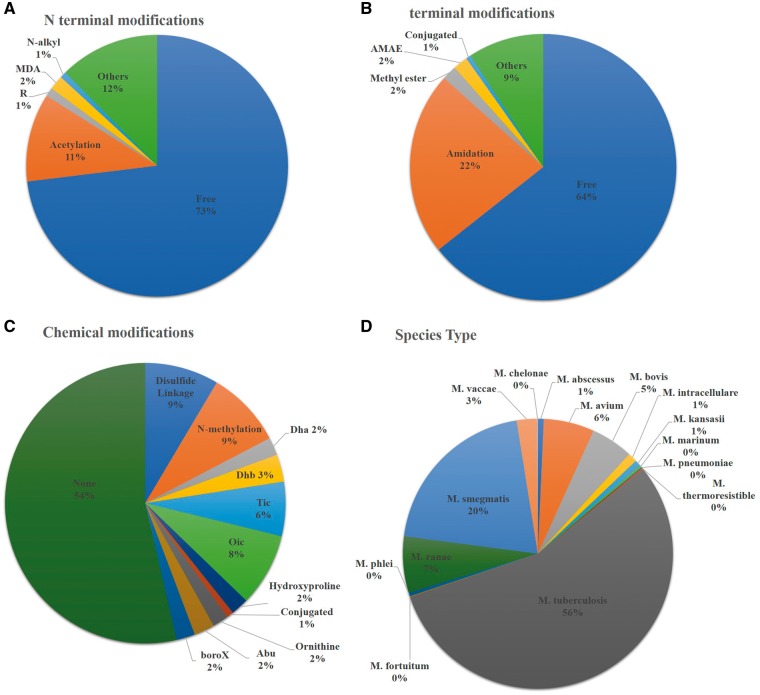
Statistical distribution of peptides in AntiTbPdb based on (**A**) N-terminal modification, (**B**) C-terminal modification, (**C**) chemical modification and (**D**) effectiveness against *Mycobacterium* species.

Researchers have done several modifications to enhance the efficacy of peptides against *Mycobacterium* species. Both end terminal (N and C terminal) as well as chemical modification such as use of non-natural amino acids, disulphide linkage and incorporation of other chemical groups (Didehydroaminobutyric acid, 2-aminobutyric acid, tetrahydroisoquinolinecarboxylic acid etc.) have been explored to see the efficacy of peptide against several *Mycobacterium* species. Most of them (550) have been studied against *M. tuberculosis* but the high cost and requirement of highly equipped biosafety level -3, enforced researchers to conduct their research using *Mycobacteriumsmegmatis.* Therefore, a large number of studies have been conducted against *M. smegmatis* (202)*.* Beside this, several other *Mycobacterium* species have been explored to see the efficacy of peptide against these species (Figure 3). We have incorporated a moonlighting browse facility, which will be very helpful to search all the peptides, which have activity against more than one *Mycobacterium* Species. For example, user can search the peptide, which has been studied against *M. tuberculosis* as well as *M. marinum* species.

The major mechanism behind killing or reducing the extent of *Mycobacterium* using specific peptide lies in the disruption of cell envelope. Most of the peptides like Human neutrophil defensins (HNPs), Protegrin-1 (PG-1) (28), Polydim (29), Pin 2 (30), Bacteriocins (31) etc. act on *Mycobacterium* by disrupting its cell envelope either by forming pore or increases permeabilization by causing membrane depolarization (32) or by disrupting cell wall biosynthetic pathway (33, 34). Beside this several anti-TB peptide perform its inhibition activity by targeting specific enzyme like Mycosin protease-1 (MycP1) (35), ClpC1 ATPase (36, 37), ClpP1P2 peptidase (38), etc.

### Comparison with other anti-microbial peptide databases

Several anti-microbial peptide databases exist, and we examined three heavily used and most updated existing databases of anti-microbial peptides i.e. APD3, DBAASP v2, CAMPR3 and observed that large number of anti-tubercular and anti-mycobacterial peptides are not covered in these databases. As compare to 542 unique peptides in AntiTbPdb around 78, 82 and 310 peptides were covered in APD3, CAMPR3 and DBASSP v2, respectively. It means two of them cover around 15% peptides maintained in AntiTbPdb. In addition, AntiTbPdb provides more information about each peptide than other resources. Though all three databases provide information about large number of anti-microbial peptides but AntiTbPdb provides more information about anti-tuberculosis and anti-mycobacterial peptides. In summary, AntiTbPdb will complement existing databases in providing tuberculosis-specific information.

## Limitations and update of AntiTbPdb

AntiTbPdb also provides structural information of peptides but there are some peptide sequences which have modification and other moieties which are beyond the scope of art of prediction techniques involved in PEPstrMOD. Beside this, there are few peptides and peptide-antibiotics, whose sequence information is not available in the literature, these peptides have been stored without sequence information. As soon as the sequence will be known, it will be updated.

The web server allows users to submit new entry of anti-mycobacterial peptide on-line by filling HTML form. However, we will confirm the validity of new entry, to maintain the quality of resource. Our team is also searching and adding new entries of anti-tubercular peptides in database from published literature. Attempts will be made to update this database regularly twice a year. 

## Discussion

Despite the huge research against *Mycobacterium*, it is still a major concern for mankind. Emergence of drug resistant strain and limitations of conventional therapy, enforced researchers to work further to fully eradicate this disease. Among the promising candidates, anti-mycobacterial peptides emerged as potential therapeutic candidates. Most of the current anti-mycobacterial peptides are derived from some natural source such as either by bacterial extraction, mycobacteriophages or from host immune cells. Peptides derived from the proteins of innate immune system such as HNPs and defensins has already been studied for their effectiveness against broad range of viruses, fungi and bacteria including *M. tuberculosis* (39). The major mechanism behind the bactericidal activity of peptides is membrane disruption through pore formation. Beside this many of the anti-tubercular peptides also act on a secondary non-membrane target, thus making it effective against *M. tuberculosis*, for e.g. HNP-1 kills *M. tuberculosis* by disrupting cell envelope as well as targeting DNA (40). One of the major reasons of resistance against drugs is thought to be the inability to penetrate cell wall and it is a well-known fact that *Mycobacterium* persist in host cells and modulate the host immune response in its own favor, therefore effective anti-TB agents must be able to penetrate through macrophages and perform effective intracellular killing without molesting other cells. In this ambience, anti-mycobacterial peptides have been proven very effective by the virtue of their affinity towards cell envelope, low immunogenicity and diverse mode of actions through interacting with secondary intracellular targets such as nucleic acid, enzymes and even organelle (37, 41, 42). *In vivo* stability, oral bioavailability, and short half-life are the major obstacle in their use as therapeutic. This has been conquered by structural constrains and introducing non-natural amino acids as well as by several chemical modifications.

Understanding of structural-functional relationship of peptides with their molecular mechanism of action, and their immune-modulating properties in infectious disease will be very useful in future drug discoveries. Thus, we anticipate that, AntiTbPdb will be a very useful resource for the researcher to design novel anti-tubercular peptide and further characterization of already existing effective anti-tubercular peptide to kick it up a notch.

## References

[1] Sharma S.K., Mohan A. (2013) Tuberculosis: from an incurable scourge to a curable disease–journey over a millennium. Indian J. Med. Res., 137, 455–493. 23640554 PMC3705655

[2] Wang J.-Y., Sun H.-Y., Wang J.-T. (2015) Nine- to twelve-month anti-tuberculosis treatment is associated with a lower recurrence rate than 6-9-month treatment in human immunodeficiency virus-infected patients: a retrospective population-based cohort study in Taiwan. PLoS One, 10, e0144136. 26633835 10.1371/journal.pone.0144136 PMC4669121

[3] Migliori G.B., Loddenkemper R., Blasi F. (2007) 125 years after Robert Koch’s discovery of the tubercle bacillus: the new XDR-TB threat. Is "science" enough to tackle the epidemic? Eur. Respir. J., 29, 423–427. 17329486 10.1183/09031936.00001307

[4] Gordon Y.J., Romanowski E.G., McDermott A.M. (2005) A review of antimicrobial peptides and their therapeutic potential as anti-infective drugs. Curr. Eye Res., 30, 505–515. 16020284 10.1080/02713680590968637 PMC1497874

[5] Ramón-García S., Mikut R., Ng C. (2013) Targeting *Mycobacterium tuberculosis* and other microbial pathogens using improved synthetic antibacterial peptides. Antimicrob. Agents Chemother., 57, 2295–2303. 23478953 10.1128/AAC.00175-13 PMC3632949

[6] Yu G., Baeder D.Y., Regoes R.R. (2016) Combination effects of antimicrobial peptides. Antimicrob. Agents Chemother., 60, 1717–1724. 26729502 10.1128/AAC.02434-15 PMC4775937

[7] Nuding S., Frasch T., Schaller M. (2014) Synergistic effects of antimicrobial peptides and antibiotics against Clostridium difficile. Antimicrob. Agents Chemother., 58, 5719–5725. 25022581 10.1128/AAC.02542-14 PMC4187972

[8] Padhi A., Sengupta M., Sengupta S. (2014) Antimicrobial peptides and proteins in mycobacterial therapy: current status and future prospects. Tuberculosis (Edinb), 94, 363–373. 24813349 10.1016/j.tube.2014.03.011

[9] Yount N.Y., Yeaman M.R. (2004) Multidimensional signatures in antimicrobial peptides. Proc. Natl. Acad. Sci. USA, 101, 7363–7368. 15118082 10.1073/pnas.0401567101 PMC409924

[10] Jemimah S., Yugandhar K., Michael Gromiha M. (2017) PROXiMATE: a database of mutant protein-protein complex thermodynamics and kinetics. Bioinformatics, 33, 2787–2788. 28498885 10.1093/bioinformatics/btx312

[11] Kumar M.D.S., Gromiha M.M. (2006) PINT: protein-protein interactions thermodynamic database. Nucleic Acids Res., 34, D195–D198. 16381844 10.1093/nar/gkj017 PMC1347380

[12] Dhanda S.K., Usmani S.S., Agrawal P. (2017) Novel in silico tools for designing peptide-based subunit vaccines and immunotherapeutics. Brief. Bioinform., 18, 467–478. 27016393 10.1093/bib/bbw025

[13] Bhalla S., Verma R., Kaur H. (2017) CancerPDF: a repository of cancer-associated peptidome found in human biofluids. Sci. Rep., 7, 1511. 28473704 10.1038/s41598-017-01633-3 PMC5431423

[14] Agrawal P., Bhalla S., Usmani S.S. (2016) CPPsite 2.0: a repository of experimentally validated cell-penetrating peptides. Nucleic Acids Res., 44, D1098–D1103. 26586798 10.1093/nar/gkv1266 PMC4702894

[15] Singh S., Chaudhary K., Dhanda S.K. (2016) SATPdb: a database of structurally annotated therapeutic peptides. Nucleic Acids Res., 44, D1119–D1126. 26527728 10.1093/nar/gkv1114 PMC4702810

[16] Usmani S.S., Bedi G., Samuel J.S. (2017) THPdb: database of FDA-approved peptide and protein therapeutics. PLoS One, 12, e0181748. 28759605 10.1371/journal.pone.0181748 PMC5536290

[17] Vita R., Overton J.A., Greenbaum J.A. (2015) The immune epitope database (IEDB) 3.0. Nucleic Acids Res., 43, D405–D412. 25300482 10.1093/nar/gku938 PMC4384014

[18] Thangakani A.M., Nagarajan R., Kumar S. (2016) CPAD, curated protein aggregation database: a repository of manually curated experimental data on protein and peptide aggregation. PLoS One, 11, e0152949. 27043825 10.1371/journal.pone.0152949 PMC4820268

[19] Wang G., Li X., Wang Z. (2016) APD3: the antimicrobial peptide database as a tool for research and education. Nucleic Acids Res., 44, D1087–D1093. 26602694 10.1093/nar/gkv1278 PMC4702905

[20] Waghu F.H., Barai R.S., Gurung P. (2016) CAMPR3: a database on sequences, structures and signatures of antimicrobial peptides. Nucleic Acids Res., 44, D1094–D1097. 26467475 10.1093/nar/gkv1051 PMC4702787

[21] Fan L., Sun J., Zhou M. (2016) DRAMP: a comprehensive data repository of antimicrobial peptides. Sci. Rep., 6, 24482. 27075512 10.1038/srep24482 PMC4830929

[22] Zhao X., Wu H., Lu H. (2013) LAMP: a database linking antimicrobial peptides. PLoS One, 8, e66557. 23825543 10.1371/journal.pone.0066557 PMC3688957

[23] Pirtskhalava M., Gabrielian A., Cruz P. (2016) DBAASP v.2: an enhanced database of structure and antimicrobial/cytotoxic activity of natural and synthetic peptides. Nucleic Acids Res., 44, D1104–D1112. 26578581 10.1093/nar/gkv1174 PMC4702840

[24] Singh S., Singh H., Tuknait A. (2015) PEPstrMOD: structure prediction of peptides containing natural, non-natural and modified residues. Biol. Direct, 10, 73. 26690490 10.1186/s13062-015-0103-4 PMC4687368

[25] Yang J., Yan R., Roy A. (2015) The I-TASSER Suite: protein structure and function prediction. Nat. Methods, 12, 7–8. 25549265 10.1038/nmeth.3213 PMC4428668

[26] Altschul S.F., Gish W., Miller W. (1990) Basic local alignment search tool. J. Mol. Biol., 215, 403–410. 2231712 10.1016/S0022-2836(05)80360-2

[27] Smith T.F., Waterman M.S. (1981) Identification of common molecular subsequences. J. Mol. Biol., 147, 195–197. 7265238 10.1016/0022-2836(81)90087-5

[28] Linde C.M., Hoffner S.E., Refai E. (2001) In vitro activity of PR-39, a proline-arginine-rich peptide, against susceptible and multi-drug-resistant *Mycobacterium tuberculosis*. J. Antimicrob. Chemother., 47, 575–580. 11328767 10.1093/jac/47.5.575

[29] das Neves R.C., Trentini M.M., de Castro e Silva J. (2016) Antimycobacterial activity of a new peptide polydim-I isolated from neotropical social wasp Polybia dimorpha. PLoS One, 11, e0149729. 26930596 10.1371/journal.pone.0149729 PMC4773228

[30] Rodríguez A., Villegas E., Montoya-Rosales A. (2014) Characterization of antibacterial and hemolytic activity of synthetic pandinin 2 variants and their inhibition against *Mycobacterium tuberculosis*. PLoS One, 9, e101742. 25019413 10.1371/journal.pone.0101742 PMC4096598

[31] Sosunov V., Mischenko V., Eruslanov B. (2007) Antimycobacterial activity of bacteriocins and their complexes with liposomes. J. Antimicrob. Chemother., 59, 919–925. 17347179 10.1093/jac/dkm053

[32] Pulido D., Torrent M., Andreu D. (2013) Two human host defense ribonucleases against mycobacteria, the eosinophil cationic protein (RNase 3) and RNase 7. Antimicrob. Agents Chemother., 57, 3797–3805. 23716047 10.1128/AAC.00428-13 PMC3719706

[33] Samuchiwal S.K., Tousif S., Singh D.K. (2014) A novel peptide interferes with *Mycobacterium tuberculosis* virulence and survival. FEBS Open Bio., 4, 735–740. 10.1016/j.fob.2014.08.001 PMC4208091 25349777

[34] Horváti K., Mezo G., Szabó N. (2009) Peptide conjugates of therapeutically used antitubercular isoniazid-design, synthesis and antimycobacterial effect. J. Pept. Sci., 15, 385–391. 19319854 10.1002/psc.1129

[35] Frasinyuk M.S., Kwiatkowski S., Wagner J.M. (2014) Pentapeptide boronic acid inhibitors of *Mycobacterium tuberculosis* MycP1 protease. Bioorg. Med. Chem. Lett., 24, 3546–3548. 24915878 10.1016/j.bmcl.2014.05.056 PMC4120117

[36] Gavrish E., Sit C.S., Cao S. (2014) Lassomycin, a ribosomally synthesized cyclic peptide, kills *Mycobacterium tuberculosis* by targeting the ATP-dependent protease ClpC1P1P2. Chem. Biol., 21, 509–518. 24684906 10.1016/j.chembiol.2014.01.014 PMC4060151

[37] Gao W., Kim J.-Y., Anderson J.R. (2015) The cyclic peptide ecumicin targeting ClpC1 is active against *Mycobacterium tuberculosis* in vivo. Antimicrob. Agents Chemother., 59, 880–889. 25421483 10.1128/AAC.04054-14 PMC4335914

[38] Akopian T., Kandror O., Tsu C. (2015) Cleavage specificity of *Mycobacterium tuberculosis* ClpP1P2 protease and identification of novel peptide substrates and boronate inhibitors with anti-bacterial activity. J. Biol. Chem., 290, 11008–11020. 25759383 10.1074/jbc.M114.625640 PMC4409261

[39] De Leon Rodriguez L.M., Kaur H., Brimble M.A. (2016) Synthesis and bioactivity of antitubercular peptides and peptidomimetics: an update. Org. Biomol. Chem., 14, 1177–1187. 26645944 10.1039/c5ob02298c

[40] Sharma S., Khuller G. (2001) DNA as the intracellular secondary target for antibacterial action of human neutrophil peptide-I against *Mycobacterium tuberculosis* H37Ra. Curr. Microbiol., 43, 74–76. 11375668 10.1007/s002840010263

[41] Abedinzadeh M., Gaeini M., Sardari S. (2015) Natural antimicrobial peptides against *Mycobacterium tuberculosis*. J. Antimicrob. Chemother., 70, 1285–1289. 25681127 10.1093/jac/dku570

[42] Khusro A., Aarti C., Agastian P. (2016) Anti-tubercular peptides: a quest of future therapeutic weapon to combat tuberculosis. Asian Pac. J. Trop. Med., 9, 1023–1034. 27890360 10.1016/j.apjtm.2016.09.005

